# Supplementary material for: An unclean slate, discrepancies between food input and recovered protein signal from experimental foodcrusts
Source: PLoS One. 2025 Aug 22;20(8):e0330195. doi: 10.1371/journal.pone.0330195 (PMC12373221; doi:10.1371/journal.pone.0330195)
Supplement: Attachment — Submitted filename: Response to reviewers.docx [file pone.0330195.s008.docx]

**Response to reviewers regarding Dekker *et al.* ‘An unclean slate, discrepancies between food input and recovered protein signal from experimental foodcrusts’**

**Reviewer #1:**

Proteomic analysis of food residues in archaeological ceramics is an emerging field which has the potential to provide us with valuable information regarding utilisation of resources and reconstruction of dietary pathways and food chains. However, progress in the field is hampered by the relative paucity of experimental archaeology studies in this field. As such, the present manuscript is a valuable contribution in the investigation of the relationship between the input resources and the subsequent formation of foodcrusts and the extraction of proteins from them and their interpretation to trace back the resources used.

The manuscript is scientifically sound, and the methodological techniques used are relevant and well-described. The conclusions drawn are broadly supported by the experiments described in the manuscript, and the results are discussed in an archaeological and interpretive context.

However, I have a few minor questions and concerns which I believe need to be addressed before the manuscript can be published.

We appreciate the kind words of reviewer 1 and hope to have successfully addressed the questions and concerns in the text below.

All line number references in the document below refer to the tracked changes version.

Materials and Methods –

Can the authors discuss the possible reasons behind the formation of the foodcrust in the rim rather than any other region? I presume this is due to the possible evaporation in the region near the rim, but I will be interested in what the authors think about it.

We agree with the reviewer that evaporation is likely an important factor in foodcrust formation. Our own observations indicate that the foodcrust forms most abundantly where the waterline recedes and this matches other anecdotal evidence that foodcrust only forms after substantial evaporation of any liquid. We have added lines 692-703 to the Discussion in order to reflect on this.

I assume the cooking simulation experiment was carried using indirect heating, i.e., the ceramics were not placed directly in a heating element. If that ist he case, can the authors please mention and discuss why that approach was chosen rather than direct heating? To me, it appears more likely that archaeological pots would have been heated directly in an open flame, and as such a direct heat would have been a better approximation of the treatment undergone by foodstuff, and hence the formation of foodcrust more representative what likely happened in an archaeological context.

We acknowledge that the reviewer is right in pointing out that direct heating would have likely been more representative of Mesolithic cooking, but the use of direct heating would have introduced many more variables and resulted in significant variation in the temperature the vessels were exposed to. For this experiment, because so little is yet known about the conditions of foodcrust formation, we preferred a more controlled approach to maximise the reproducibility and comparability of our results. We have added lines 153-159 to the Method section to elaborate on our experimental design.

Can the authors please provide descriptions of the burial conditions (namely substrate/soil, approximate temperature, moisture content vs dryness of the substrate etc.) at York Experimental Archaeological Research Centre?

We have added additional information regarding the burial conditions in lines 181-185. Unfortunately, no previous data regarding moisture content was available from the YEAR centre. We discussed the possibility of acquiring this data with the YEAR centre, but it seems this would require a substantial amount of additional work. Considering the reporting of contextual data from publications concerning other similar experiments [(Bondetti et al. 2021; Evans et al. 2024)](https://paperpile.com/c/nNCPOj/HZ0v+vet9), we hope to have provided sufficient data to ensure the comparability of our results.

Additionally, in order to obtain representative data this would need to happen during the same months as the burial experiment. As the burial experiment was conducted during August and July, we would not be able to submit the revised manuscript in time.

Protein data analysis

Can the authors please search the mass spectrometry data against a database of common contaminants like cRap? This will be helpful in identifying the prevalence of contaminant proteins and any possible misidentitication regarding them, which can be a potential explanantion for the protein A0A3Q1LL35, as the authors mention in line 463.

We had indeed used the in-built cRAP database of common contaminants to check for contaminants, but forgot to include this in the manuscript. Our apologies, the text has now been updated (lines 261-263). Additionally, we have included some further metadata from MetaMorpheus regarding used databases, identified PSMs and proteins in SI 3.

Discussion

Disentangling mixtures & taxonomic representativeness

Can the authors please discuss the proteins recovered after various cooking events in some greater detail by comparing and contrasting between the proteins recovered after various extraction, with specific focus on proteins recovered after the first cooking event? It appears to me that the first cooking event is perhaps most likely to estimate the conditions of archaeological ceramics- to me, it appears unlikely that the same pot was used for multiple cooking events without scrubbing off any visible charred residue. If that is the case, the residues after the first cooking event are probably most relevant for interpretation of archaeological data, and as such need to discussed in greater detail.

We have added lines 339-352, 374-437 and 492-511 to expand our discussion on the recovery of particular proteins, as well as additional analysis regarding characteristics of the recovered proteins. In short, we compare the most intense proteins from after the first cooking event to the proteins persisting throughout all cooking events, we show the hydrophobicity of the identified fractions of the proteins and we compare the occupancy of PTMs of the most intense proteins between the cooking events.

However, we would like to add that the notion that it is unlikely to use the same pot for multiple cooking events without scrubbing off residue, is an assumption. The amount and type of residue that formed after our first cooking event is less similar to foodcrusts observed on archaeological material, than the foodcrusts that formed after later events.

Can the authors please add a small paragraph comparing the nature/type of the various proteins recovered from the foodcrust with the type of proteins recovered in (i) other studies involving proteomics on archaeological samples where evidence of similar foodstuff processing has been recovered (ii) other experimental studies involving recovery of proteins from ceramic matrix and involving similar foodstuff?

The authors go into this in some detail re Evans et al. (line 501), but I believe a similar comparison involving additional studies (and comments on the differences in the types of proteins extracted) will be beneficial. This is particularly relevant since some prior studies have shown the recovery of muscle/blood proteins as evidence of animal processing, contrasting with the disappearance of the Bos taurus proteins here after burial. Is it possible that the nature of the substrate (foodcrust vs calcified deposits vs ceramic matrix) can affect the protein preservation and hence the interpretation of resource utilisation?

In order to better contextualise our results in the wider literature we have added lines 647-662 to compare our results with an additional number of previous studies on experimental and archaeological organic residues.

**Reviewer #2:**

In this manuscript, Dekker et al. describe an experimental archaeological study to create foodcrusts in clay vessels and characterize their protein content over cooking and a variety of input conditions. This project is very well planned and executed by the authors. I'm impressed with the organization of this relatively complex set of experiments.

Line 88-89: "one lipid-rich, one protein-rich and one carbohydrate-rich to mix" Add material or food after carbohydrate-rich.

Added ‘foodstuff’

Line 182: How were the foodcrusts homogenized?

Replaced ‘homogenised’ with ‘vortexed’ to clarify the used procedure.

Line 234-237: For G-PTM-D, at minimum include the number of PTMs included and the PTM groups. If only specific ones were included, add them here or in the supplemental.

We have added the included PTM groups and number of PTMs, now at line 246-251. Additionally, the exact list of PTMs can be found in SI 3, Task2GptmdTaskconfig.toml.

Line 240-242: Include the download dates of the Uniprot databases.

Added the download dates of the used UniProt databases.

Figure 3: Unless the protein input is a continuous measurement, use factor for the scale instead of number.

We presume the reviewer referred to Figure 4 rather than Figure 3? The scale in Figure 4 for total protein content was changed to factor.

In case Figure 3 was intended, the experimental vessels were filled with foodstuffs according to the exact percentages listed in the figure.

Table 1: Include a column of the inputs for each cooking event. It may abe difficult to cross reference figure 1 with table 1 in the published version.

Added columns indicating the % beetroot, % salmon and % salmon.

Figure 4: The lines between the boxes may not be clear if the figure is small at the final publication.

We have added additional space between the different boxplots.

Line 333-338: Use the protein name instead of the Uniprot accession or put the accession in parentheses. It is hard to tell what protein it is from the accession alone.

We have updated all references to specific proteins (except for A0A0J8B2U8, the uncharacterised protein) to include the full name and the accession number in parentheses.

Line 534 and 535: Should Fig. 6 be Fig. 7? This seems to be about the foodcrust size and not tissue specificity.

We would like to thank the reviewer for spotting this mistake, we have amended it.

Line 538-539: "Suggesting that the presence of such a fat-rich food is not necessarily a driver of foodcrust formation" I think it may be worth connecting this result to the use of fats in reducing sticking in modern cooking.

We agree with the reviewer that there is an interesting connection between foodcrust formation and modern cooking. To expand on this suggestion we have added lines X to incorporate some insights from studies on industrial ‘fouling’.

Line 541: Confirm that this should be Fig. 5 and not another figure. It could be figure 2 or 7.

We have updated the reference to the right figure here.

SI 5: Use a colorblind accessible gradient instead of the Red-Green one.

We have changed the gradient of SI 5 to red-blue for improved visibility.
